# Replication, Pathogenesis and Transmission of Pandemic (H1N1) 2009 Virus in Non-Immune Pigs

**DOI:** 10.1371/journal.pone.0009068

**Published:** 2010-02-05

**Authors:** Sharon M. Brookes, Alejandro Núñez, Bhudipa Choudhury, Mikhail Matrosovich, Stephen C. Essen, Derek Clifford, Marek J. Slomka, Gaëlle Kuntz-Simon, Fanny Garcon, Bethany Nash, Amanda Hanna, Peter M. H. Heegaard, Stéphane Quéguiner, Chiara Chiapponi, Michel Bublot, Jaime Maldonado Garcia, Rebecca Gardner, Emanuela Foni, Willie Loeffen, Lars Larsen, Kristien Van Reeth, Jill Banks, Richard M. Irvine, Ian H. Brown

**Affiliations:** 1 Veterinary Laboratories Agency-Weybridge, EU/OIE/FAO Reference Laboratory for Avian Influenza and Newcastle Disease, Addlestone, Surrey, United Kingdom; 2 University of Ghent, Merelbeke, Belgium; 3 Agence Française de Sécurité Sanitaire des Aliments, LERAPP, Unité Virologie Immunologie Porcines, Zoopôle Les Croix, Ploufragan, France; 4 Central Veterinary Institute of Wageningen UR, Lelystad, The Netherlands; 5 Istituto Zooprofilattico Sperimentale Lombardia ed Emilia Romagna, Sezione di Parma, Parma, Italy; 6 National Veterinary Institute, Technical University of Denmark, København, Denmark; 7 Institute of Virology, Philipps University, Marburg, Germany; 8 Merial, S.A.S., R&D, Lyon, France; 9 Laboratorios HIPRA, Amer Girona, Spain; 10 OFFLU, World Organisation for Animal Health, Paris, France; University of Georgia, United States of America

## Abstract

The declaration of the human influenza A pandemic (H1N1) 2009 (H1N1/09) raised important questions, including origin and host range [1], [2]. Two of the three pandemics in the last century resulted in the spread of virus to pigs (H1N1, 1918; H3N2, 1968) with subsequent independent establishment and evolution within swine worldwide [3]. A key public and veterinary health consideration in the context of the evolving pandemic is whether the H1N1/09 virus could become established in pig populations [4]. We performed an infection and transmission study in pigs with A/California/07/09. In combination, clinical, pathological, modified influenza A matrix gene real time RT-PCR and viral genomic analyses have shown that infection results in the induction of clinical signs, viral pathogenesis restricted to the respiratory tract, infection dynamics consistent with endemic strains of influenza A in pigs, virus transmissibility between pigs and virus-host adaptation events. Our results demonstrate that extant H1N1/09 is fully capable of becoming established in global pig populations. We also show the roles of viral receptor specificity in both transmission and tissue tropism. Remarkably, following direct inoculation of pigs with virus quasispecies differing by amino acid substitutions in the haemagglutinin receptor-binding site, only virus with aspartic acid at position 225 (225D) was detected in nasal secretions of contact infected pigs. In contrast, in lower respiratory tract samples from directly inoculated pigs, with clearly demonstrable pulmonary pathology, there was apparent selection of a virus variant with glycine (225G). These findings provide potential clues to the existence and biological significance of viral receptor-binding variants with 225D and 225G during the 1918 pandemic [5].

## Introduction

In April 2009, an H1N1 virus was detected in humans [1] that was described as putatively of swine origin since seven of the eight gene segments originated from historical swine influenza viruses [2]. Further cases of infection of humans with this virus in the absence of direct contact with pigs were rapidly described together with proven human-to-human transmission. Although the virus ostensibly derived from pig populations, the consequences of infection in swine were unknown. The role of pigs in the epidemiology of influenza has been well described [3], although their direct involvement in the genesis of a pandemic influenza virus strain has never been demonstrated. Following reverse zoonosis (spread of virus from humans to animals) into pig populations, viruses are subjected to different selection pressures and evolve in distinct lineages, enabling clear separation from their counterpart strains circulating in humans. During the last ten years, substantial changes have been noted in the epidemiology of influenza in pigs, particularly with reference to significant geographical variations in terms of the virus subtypes and their associated genotypes. The pandemic (H1N1) 2009 virus shows genetic and antigenic distance in the major glycoproteins of the virus compared to contemporaneous swine strains [2]. Therefore, important questions are raised regarding pigs given their well-established linkage in the evolution and ecology of influenza viruses.

In this study, using an established infection and transmission model [6], [7], we address the outcomes, infection dynamics and pathogenesis of H1N1/09 virus infection in non-immune pigs.

## Results

### Clinical Signs

Clinical signs were detectable in all of the pigs (100% morbidity) infected directly (INF) via the intranasal route or contact exposed (TC) (Methods), with variations in the range, pattern and severity between individuals although all pigs apparently recovered. The signs induced remained typical of influenza A infections in pigs (see Text S1 and Table S1).

**Figure 1:**
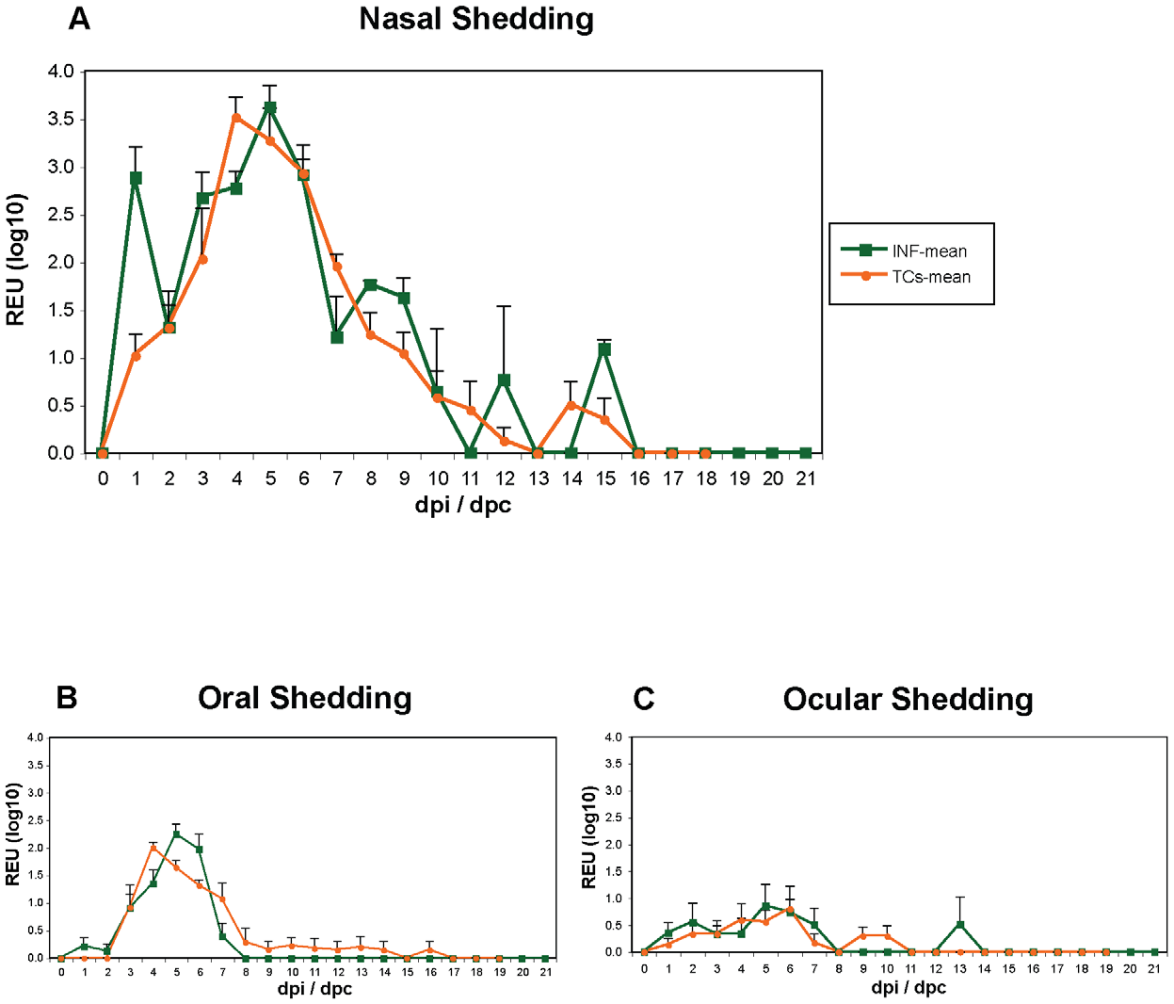
Semi-quantitative H1N1/09 virus shedding. Shedding from infected (INF) and transmission cycle (TC) pigs from the nasal (a), oral (b) and ocular (c) routes. Shedding expressed as (REU log_10_; mean + SE). Relative equivalent units (REU) represent an equivalent viral infectivity titre related to RRT-PCR threshold cycle (Ct) values. dpi/dpc refers to days post infection/contact.

The onset of clinical disease was characterised by elevations in rectal temperature and serous, bilateral nasal discharge between 1–3 days post-infection (dpi), generally coincident with the onset and maintenance of nasal virus shedding (Fig. 1A). In pigs that were INF, peak rectal temperatures in individual animals (>39.5°C) ranged from dpi 1–5 (modal value dpi 2), with mean rectal temperatures peaking from dpi 3–8. Biphasic rectal temperatures profiles were observed in some animals (3/4 infected pigs remaining from dpi 5 onwards; 5/8 in-contacts), interspersed by a substantial temperature reduction (≥1°C) in some individual animals below the mean control animal rectal temperature (38.0°C). Highly significant differences between the overall mean rectal temperatures of the INF, TC and control animals (P<0.001) were also observed. Furthermore, variations were apparent in the overall peak and intervals to peak rectal temperatures recorded for the in-contact pigs, the mean clinical scores (per day post-exposure) between INF and TC pigs, and in daily live weight gain comparing INF, TC and control pigs (Text S1 and Figures S2 and S3).

Other observed clinical signs included bilateral serous ocular discharge, coughing with an increased respiratory rate, lethargy and inappetence. The observed initial serous nasal discharge progressed to be mucoid and then muco-purulent in nature by dpi 4 in the remaining infected and contact exposed pigs, with discharge resolving in survivors by dpi 10–16.

### Virus Shedding

Shedding was measured in all samples through the detection of viral RNA in quantitative real-time RT-PCR. By the use of a standard curve (on each test plate) generated from a dilution series of infectious H1N1/09 virus quantitated in standard infectivity titrations, we were able to determine the relative equivalent unit (REU) from viral RNA based upon the cycle threshold (CT) value obtained for each sample. Virus shedding was detected from all of the INF pigs, mainly via the nasal route (Fig. 1). Peak nasal shedding (3–4 REU log_10_) occurred between 4–6 dpi, with intermittent, low level shedding (1–2 REU log_10_) from dpi 10–15, and ceased from dpi 16 (Fig. 1A).

All uninfected pigs exposed by contact with infected pigs during the four successive transmission cycles (see Methods: Groups TC1, TC2, TC3 and TC4) developed broadly similar trajectories of infection to the directly infected pigs (Figure S4). Furthermore, irrespective of exposure mode, all pigs shed similar amounts of virus from the nasal route (Fig. 1A). In contrast, overall lower levels of shedding, but with similar trajectories to that of the nasal cavity were detected from the oral (Fig. 1B) and ocular (Fig. 1C) routes (individual pigs peak range for either route was 1.5–3.0 REU log_10_). Rectal shedding was rare, detected on just three occasions (peak 2.3 REU log_10_) from two infected animals (INF, 3245 and 3261) only (at dpi 2–5). In-contact pigs (TC1-TC4) developed similar infection and nasal (Figure S4), oral and ocular shedding profiles to INF pigs (Fig. 1). However, variations were evident in the intervals to both the onset and peak of nasal shedding between TC groups (see Text S1).

### Transmission Cycle Shedding Patterns

The onset of nasal shedding was detected from one day post-contact (dpc 1) for all pigs in Groups TC1, TC2, and TC3, and from dpc 2 during TC4. Variations were also evident between contact transmission groups in the interval to and duration of peak nasal shedding, and the total duration of nasal shedding (Figure S4). During TC1, nasal shedding from the pair of exposed pigs peaked at dpc 3–6, with an overall duration of 7 days and 10 days respectively. Similarly, during TC2, peak nasal shedding was detected at dpc 6, with no further shedding after dpc 12–13. Overall nasal shedding from the exposed pigs during TC3 was detected for longer (dpc 14). However, the interval to peak nasal shedding was similar to that in TC1 and TC2 (dpc 4–6). Peak nasal shedding was detected at dpc 3–4 during TC4, with no detectable shedding via the nasal cavity from dpc 9.

These data also show that the contact period required for successful transmission of virus to both pigs, as determined by the detection of nasal shedding and a mean REU >1.5 log_10_ (at which point the TC was initiated), was 72 hours for TC1, TC2 and TC4. Interestingly, during TC3, a period of four days was required for these criteria to be fulfilled. In addition, during TC1 and TC4, peak shedding was 2–3 days earlier than in TC2 and TC3. However, overall the interval to onset and the duration of peak shedding was broadly similar in the infected and all of the contact exposed (TC1-TC4) pigs (Figure S4, panel F).

### Serology

At the start of the study the seronegative status of all animals was confirmed by haemagglutination inhibition (HI) test using four swine influenza virus subtypes (classical H1N1, avian-like swine H1N1, swine H1N2 and human-like swine H3N2) and A/California/07/2009(H1N1)v. During the study, samples were tested using A/California/07/2009(H1N1)v only. Two of four INF animals seroconverted by dpi 7, with substantial homologous HI titres (80 and 320) detected from the two pigs (3245, 3261) that remained in the study beyond that point. The eight TC animals seroconverted between 10–14 dpc, with peak HI titres in the range 40–640 from 14–18 dpc.

### Acute Phase Protein (APP) Responses

Selected APP responses were measured in serum samples collected from infected animals, specifically C-reactive protein and haptoglobin. Infection kinetics were supported by increases above the acute phase response threshold [8], [9] in the levels of both APPs over time (dpi/dpc) in all of the infected pigs (Figure S5). The C-reactive protein levels peaked at dpi/dpc 4 with more protracted responses detected after dpi/dpc 7 in one of the remaining infected pigs (3261) and the in-contact animals. The haptoglobin responses were more protracted, peaking at dpi/dpc 9–11 in both infected and TC pigs.

### Pathology

**Figure 2:**
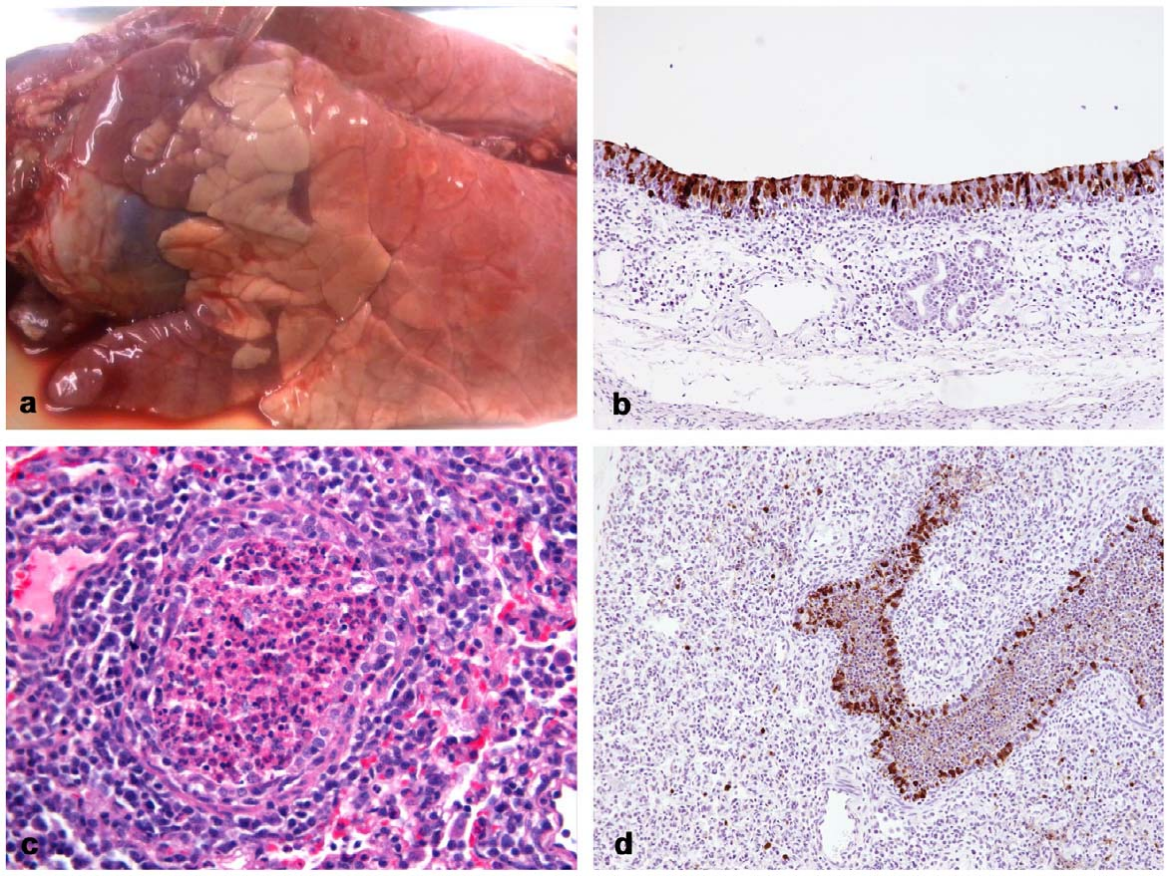
Pathological changes (a,c) and immunohistochemical detection of viral antigen. **a**. Lung. In-contact animal (TC4), 11 dpc. Multiple dark red areas of cranioventral pulmonary consolidation in the cranial and middle lung lobes. **b**. Nasal mucosa, 2 dpi. Influenza A nucleoprotein (brown) in numerous respiratory epithelial cells 20x. **c**. Lung, 7 dpi. Marked attenuation of bronchiolar epithelium due to epithelial cell necrosis, bronchiolar plug and peribronchiolar lymphohistiocytic infiltration. Haematoxylin and eosin 40x. **d**. Lung, 4 dpi. Viral nucleoprotein in abundant bronchiolar epithelial cells and cellular debris in the bronchiolar lumen and in several pulmonary alveolar macrophages 20x.

Pathological changes in infected pigs (including by contact exposure) were restricted to the respiratory tract and associated lymphoid tissues. At 3 and 4 dpi or dpc, pulmonary lesions comprised multifocal, cranioventral, lobular consolidation, increasing in number and extension at dpi/dpc 7, when the cranial and middle lung lobes appeared almost entirely consolidated (Fig. 2A and Text S1). For full details of histopathological and immunohistochemistry (IHC) findings see Text. S1 In summary histopathology revealed moderate to severe necrotising bronchiolitis, atelectasis and alveolitis followed by hyperplasia of airway epithelium as regeneration occurred in some lobules from day 7 which resolved by day 17 (Fig. 2C). The pigs were derived from a ‘PRRSV free’ source and the histopathological findings were not considered to indicate the presence of other pathogens. Furthermore, we have shown that live virus (mediated through virus re-isolation), viral antigen (detected using IHC) and viral RNA (detected by modified influenza A Matrix gene real time RT-PCR) were detectable in respiratory and associated lymphoid tissues from dpi 1–7 and dpc 11, but was not detected in examples of consumer commodities such as meat (*longissimus dorsi* and *biceps femoris*) or viscera (Table 1). This is in addition to the absence of viral RNA in plasma samples collected from directly infected animals and contact exposed pigs, consistent with no detectable viraemia.

**Table 1:** Qualitative results for the detection of virus in tissues collected from challenged or contact exposed pigs by virus re-isolation, immunohistochemistry (IHC) and RRT- PCR.

Pig ID	PME	Group	Turbinate	Naso-pharynx	Thoracic Trachea	Lung lobes				Lymph Nodes*
						Cranial	Mid	Caudal	Accessory	
3242	Day 3	Mock	−/−/−	−/−/−	−/−/−	−/−/−	−/−/−	−/−/−	−/−/−	−/−
3249	dpi 2	INF	+/+/+	+/−/+	−/−/−	−/−/−	−/−/−	−/−/−	−/−/−	−/−
3252	dpi 2	INF	+/+/+	+/−/+	+/−/+	−/−/−	+/+/+	+/−/+	+/−/+	−/+
3246	dpi 3	INF	+/+/+	−/−/+	+/−/−	+/−/+	+/+/+	+/−/+	−/−/+	−/−
3243	dpi 4	INF	+/+/+	+/−/+	+/+/+	+/+/+	+/+/+	+/+/+	+/+/+	−/+
3260	dpi 7	INF	+/+/+	−/+/−	+/+/+	+/+/+	+/+/−	+/+/+	+/+/+	−/+
3244	dpi 7	INF	+/−/+	+/−/−	−/−/−	−/+/+	+/+/−	−/+/+	+/+/+	−/+
3257	dpc 11	TC4	−/−/+	−/−/−	−/−/−	−/−/+	−/−/−	−/+***/+	−/−/−	−/−

−/−/−  =  Virus re-isolation/IHC/PCR.

* Lymph nodes, IHC: Superficial inguinal, lateral retropharyngeal**, medial retropharyngeal, right, middle and left tracheobronchial**, mediastinal and mesenteric (Virus re-isolation and PCR only).

Other: Commodities and offal - spleen, liver, kidney and ileum plus muscle (*longissimus dorsi* and *biceps femoris*) were all negative when tested by virus re-isolation & PCR.

*** Only in cell debris in lumen of three bronchioles (no bronchial or bronchiolar epithelial or macrophage immunolabelling).

Shaded rows represent a mock-infected control animal from the equivalent of dpi 3 and a transmission cycle (TC4) animal at 11 days post-contact (dpc) representing for comparative purposes the closest animal to the direct infected group PMEs.

### Sequencing and Genetic Analyses

Full genome sequence analysis was conducted for selected nasal swab RNA samples from four infected animals (3244, 3245, 3260, 3261) and both contact exposed pigs in each TC group between days 3–6 (dpi/dpc). The lowest Ct values were selected representing a REU log_10_ range of 2.3–4.1 (Table 2). There were no significant deviations from the inoculum sequence observed for all the genes encoding internal proteins and the neuraminidase gene in either directly infected or TC animals. No molecular markers for pathogenicity (e.g. PB2 E627K) were detected. Haemagglutinin (HA1) gene mutations observed throughout the study are described in Table 2 (see Text S1).

**Table 2:** HA1 locations at which amino acid changes were observed in directly infected and contact pigs.

Animal status	Pig ID	HA1		REU (log_10_)
	(dpi/dpc)	Location		
		225	226	
Inoculum:	Not applicable	X* (D/G)	X (R/Q)	
**Infected:**				
	3261 (3 dpi)	X (D/G)	Q	3.37
	3261 (4 dpi)	X (D/G)	Q	3.43
	3244 (5 dpi)	X (D/G)	Q	3.60
	3245 (5 dpi)	-	-	3.91
	3260 (5 dpi)	X (D/G)	Q	2.88
	3261 (5 dpi)	X (D/G)	Q	4.12
	3261 (6 dpi)	X (D/G)	Q	3.32
**In-contacts:**				
TC1	3240 (3 dpc)	*-*	*-*	3.66
TC1	3254 (3 dpc)	D	Q	4.16
TC2	3250 (5 dpc)	-	-	3.36
TC2	3256 (6 dpc)	D	Q	3.91
TC3	3258 (5 dpc)	D	Q	2.33
TC3	3259 (5 dpc)	D	Q	2.31
TC4	3247(5 dpc)	-	-	2.93
TC4	3257 (4 dpc)	D	Q	3.41
**Infected:** Middle lung lobe				
	3253 (1 dpi)	X (D/G)	Q	4.38
	3252 (2 dpi)	X (D/G)	Q	3.88
	3243 (4 dpi)	G	Q	6.83
	3260 (7 dpi)	G	Q	6.21

Virus derived from nasal swabs unless otherwise stated. H3 numbering [ref. 27] is used in the table and throughout the text. Where a mixed population was observed this is represented by an X with the possible mixture of amino acids described in parentheses. Dash indicates sequence data not available. Semi-quantitative nasal H1N1/09v virus shedding (REU log_10_).

The HA sequence data for the inoculum (egg passage 5) was identical to the published sequence for egg grown (egg passage 2) isolate A/California/07/2009(H1N1)v (GenBank accession: FJ969540) containing a mixture of two amino acids at positions 225 and 226 (H3 numbering), D/G and R/Q respectively. Upon infection in pigs, 226Q was selected, but 225D/G remained present. For contact animals, 226Q remained and, in addition, 225D was selected for (Table 2). HA1 sequencing was also conducted on a small subset of middle lung lobe tissue samples from directly infected pigs in order to assess variation in receptor tropism and possible relationship to pathogenesis. Of the four middle lung lobe samples analysed, all four had 226Q. Two samples (ex INF pigs dpi 1 and dpi 2) showed a dual population of 225D/G, whilst the two other samples (ex INF pigs dpi 4 and dpi 7) showed 225G (Table 2, Figure S6). Furthermore, it is interesting to note that the latter samples from INF pigs containing 225G in HA1 also had REU's >6.0 (Table 2) suggestive of efficient replication at this site.

## Discussion

In this study we have shown that pigs can be readily infected with pandemic (H1N1) 2009 virus (A/California/07/09), resulting in the induction of clinical signs, viral pathogenesis restricted to the respiratory tract, infection dynamics consistent with endemic strains of influenza A in pigs, virus transmissibility between pigs (four cycles) and virus-host adaptation events.

The type and pattern of clinical signs and induced host responses were consistent with those typical of swine influenza virus [10], including the infection dynamics, characterised by shedding from the upper respiratory tract. Rectal shedding was only rarely detected potentially reflecting a lack of tropism for replication in the alimentary tract (Table 1) that was further supported by the lack of immunolabelled enterocytes in the ileum (note the possibility of contamination of the perineum with nasal discharge cannot be excluded due the natural inquisitive and social behaviour of pigs). In addition, the onset of clinical disease was preceded by elevations in rectal temperature, and biphasic rectal temperature profiles were observed in some animals (3/4 infected pigs remaining from dpi 5 onwards; 5/8 in-contacts), a clinical feature often observed during influenza infections [11]–[13]. The measurement of selected APP responses provided further confirmation of infection dynamics. It is recognised that the porcine APP response to infection is typically characterised by elevations in serum levels of C-reactive protein as the major APP [14]. Our data supports this observation, and furthermore we have shown that C-reactive protein responses are higher in infected pigs than in TC1-TC4 pigs (Figure S5), and were more elevated in the infected and TC1 animals than the later transmission cycles.

Furthermore, our results clearly demonstrate that extant H1N1/09 is fully capable of becoming established in global pig populations. We have also shown that a 72-hour contact period within the first six days of infection is sufficient to establish virus spread in an experimental setting. We have used determinations of precise levels of viral RNA related to approximations of infectious virus which also enabled the analysis of large datasets derived from intensive sampling. However, further study would be required to refine the transmission window, and it is also important to note that events may differ in a field setting. For example, the observed levels of morbidity and mortality may differ depending on the role played by other inter-current infections and/or pig husbandry and management factors that could also result in more severe economic impacts to the swine industry. Interestingly, the limited natural occurrences of early field infections of pigs were relatively mild [15]–[17]. Similarly, whilst infection dynamics in a field setting will be different through availability and numbers of both susceptible and infected pigs, it could be reasonably expected that H1N1/09 will transmit efficiently in immunologically naïve farmed pig populations.

The pathogenesis described in this study is consistent with that of infections with endemic swine influenza viruses [10], [18], [19]. Our results with H1N1/09 were more severe than those obtained in mini-pigs [20], but similar to experiments performed in 10-week-old commercial pigs [21]. This variability may be related to virus strain, but more possibly due to breed of pig with this study using a breed commonly reared on a commercial scale. Overall, clinical signs and pathology were of much greater severity than those caused by infections with avian influenza viruses, such as highly pathogenic H5N1 or low pathogenic viruses of different subtypes, in pigs infected experimentally [22]–[24]. Furthermore they were broadly similar to those in small mammals infected with pandemic (H1N1) 2009 virus [20], [25]–[26]. Whilst we used pigs sourced from a high health herd and the histopathological findings did not indicate the presence of other pathogens, we did not further exclude intercurrent infections other than by close reference to the control pigs.

In this study we observed an apparent host selection within the H1N1/09 population based upon the haemagglutinin (HA1) gene as the virus replicated in directly infected pigs and transmitted to contact exposed pigs (Table 2). Influenza virus HA is the surface protein involved in binding host cell sialic acid in order to initiate infection, and the specificity of the HA binding depends on the virus and host species, with human and swine viruses preferentially binding α2,6-linked sialyl receptors whereas avian viruses bind α2,3-linked sialyl receptors [reviewed in ref 27].

For technical reasons we used egg-grown A/California/07/2009 (H1N1)v at passage level 5 for the direct infection of pigs. The HA gene sequencing of virus inoculum revealed that it differs from the original virus, A/California/07/2009 (GenBank accession numbers: FJ966974, FJ969540 and FJ981613)[2] through the presence of a mixture of two amino acids at HA positions 225 and 226 (H3 numbering, [27]), D/G and R/Q, respectively. We assume that the inoculum was a mixture of quasispecies of a wild type virus (HA, 225D/226Q) [2] and at least two egg-derived mutants (HA, 225G and HA, 226R). The mutation Q226R has been observed previously in egg-adapted seasonal human H1N1 viruses [28], [29]. Isolates with 226R grow well *in vitro*, but are thought to be severely impaired *in vivo* [29]. The data presented here supports this hypothesis, since the 226R variant was absent from all viruses examined from infected pigs (Table 2).

A mutation G225D/E in the HA of H1N1 viruses is known to be important for the alteration of the viral receptor specificity during adaptation of avian viruses to humans and pigs [5], [30], [31]. Interestingly, studies with the 1918 pandemic H1N1 virus describe it as circulating as a mixture of variants with either 225D or 225G [5], but the biological significance of this heterogeneity remains unknown. Both variants infected ferrets, but transmission of the variant with 225G was not as efficient as for variant 225D [29]. Our data for H1N1/09 are consistent with this finding in that all viruses detected in nasal secretions from the contact exposed pigs (TC1-TC4) possessed 225D (Table 2), indicating that it appears more transmissible than variant 225G.

Glycine at HA position 225 of human and swine H1N1 viruses enhances virus binding to α2,3 receptors without markedly compromising ability to bind to α2,6 receptors [27], [30], [31]. Although non-egg-adapted swine and seasonal human H1N1 viruses typically display 225D/E [27], [30], here we show possible selection of the 225G in affected middle lung lobe tissue from a small subset of samples from directly infected pigs, potentially indicative of different selection pressure within this compartment compared to cells of the upper respiratory tract (Table 2, Figure S6) even though cells of the lower respiratory tract of pigs express both α2,3 and α2,6 receptors [4]. These findings demonstrate the roles of viral receptor specificity in both transmission and tissue tropism providing potential clues to the existence and biological significance of viral receptor-binding variants with 225D and 225G during the 1918 pandemic [5].

We have shown that pigs are susceptible to infection with H1N1/09 and incursion from infected humans to the farmed pig population appears likely. However, the impact of such transmission will be compounded by a number of variables that we were not able to address in the current study, such as prior immunity to H1 influenza viruses, age of animal, husbandry factors, including production type, and intercurrent disease. Close monitoring of global pig populations is required if the dynamic is to be fully understood, as is further virus evolution to allow assessment of human and veterinary health risks.

Our results on different selective pressures in the upper and lower respiratory epithelium in pigs provide further insights into host selection preferences and may suggest possible differences in relation to transmissibility and pathogenesis within the same animal. In particular, this finding prompts further studies on the enigma of co-circulation of two receptor-binding variants of the 1918 pandemic virus and on their relative role in pathogenesis.

## Methods

This work was approved by the VLA ethics committee for animal studies and was carried out in accordance with the UK 1986 Animal Scientific Procedure Act and VLA code of practice for performance of scientific studies using animals (available on request). Furthermore, these guidelines meet the requirement for the European Directive for Animal Experimentation (86/609).

### Study Design

The infection and transmission study comprised twenty-two Landrace hybrid pigs, aged 4–5 weeks old at the outset, randomly allocated to six separate study groups (Figure S1). All of the pigs were sourced from a high health status herd (PRRS virus free) and were shown to be both influenza A virus and antibody (subtypes H1N1, H1N2, H3N2) negative by Matrix (M) gene real time RT-PCR (described below) and haemagglutination inhibition (HI) assays [32] respectively prior to the start of the study. Infection was initially established in a group of eleven pigs by intra-nasal inoculation with a total dose of 10^5.8^ EID_50_ A/California/07/2009(H1N1)v, delivered in a final volume of 2 ml per nostril, using a mucosal atomisation device (MAD® Nasal, Wolfe Tory Medical, Inc.) to mimic aerogenous infection. The virus strain (A/California/07/2009(H1N1v) was propagated in embryonated fowls' eggs (passage history, egg passage 5).

In addition to the group of infected pigs (INF), four successive transmission cycles (TC1-TC4) were established between infected and naïve pigs at monitored intervals (once sustained nasal shedding from both of the pigs in each cycle was confirmed). TC1 was established by placing two naïve contact exposed pigs with infected pigs at dpi 2. With nasal shedding detected from the pair of TC1 pigs, they were removed to a separate room to establish TC2, by infection of a second pair of naïve pigs. Two further transmission cycles were then established between successive pairs of infected and naïve, contact exposed pigs (TC3, TC4). Three uninfected pigs were maintained as a control group (Controls), two of which were mock-inoculated and one was non-inoculated. The mock-inoculated pigs were challenged by the intra-nasal route using allantoic fluid suspended and delivered in a final volume of 2 ml PBS per nostril, using the same method as the infected pigs.

### Clinical Measurements and Sampling

Each day throughout the duration of the study (25 days) clinical parameters were assessed by veterinary inspections, with swab samples (nasal, ocular, oral and rectal) collected. The swabs were then immediately tested by a modified influenza A M gene real-time RT (RRT)-PCR assay (described below), enabling confirmation of pig infection and shedding status (Fig. 1 and Figure S4). In addition to the daily procedures and sampling conducted for each of the directly infected pigs (INF), control animals (Controls) were swabbed on three occasions, equivalent to dpi 0, 6 and 20. The same types of swab sample were also collected on a daily basis from each contact exposed pig (TC1-4) from the first day of their respective transmission cycles and processed daily for M gene RRT-PCR testing.

### Blood Samples and Assays

Blood samples were collected by jugular venepuncture from all of the pigs prior to the start of the study, and from the directly infected pigs up to dpi 4, and again at dpi 7 for assessment of viraemia, acute phase protein responses and antibody production (described below). Blood samples for were then collected twice weekly from infected pigs, and the contact exposed pigs (TC1-TC4) as they were recruited to the study during the successive transmission cycles, with collection of terminal blood samples from all remaining pigs prior to euthanasia (Figure S5).

### Haemagglutination Inhibition (HI) Assay

Haemagglutinin specific antibodies were detected in pig sera using HI tests performed according to standard methods [32] with A/California/07/09 as antigen.

### Acute Phase Protein (APP) Responses

C-reactive protein and haptoglobin responses were measured in serum as previously described [8], [9] using a commercially available ELISA assay, according to manufacturers instructions (Tridelta Development Ltd., Kildare, Ireland) (see Protocol S1).

### Post-Mortem Examination (PME)

At defined intervals infected pigs were removed for post-mortem examination, with systematic recording of macroscopic findings and collection of a range of tissues for histopathology and virus detection assays (Table 1). Pairs of infected pigs (INF) were subject to PME on dpi 2, 3, 4 and 7, with one infected pig PME on each of dpi 1, dpi 17 and dpi 21. Further PMEs were performed on contact exposed pigs on dpi 21 and dpi 25 (Figure S1). A full range of tissue specimens was collected, with selected fresh tissue samples (Table 1) stored at −70°C. Samples collected for histopathology and IHC were stored in 10% (v/v) phosphate-buffered formalin pending final processing (described below) and analysis.

### Virus Detection in Tissues

Virus detection (Table 1) was performed by IHC, M gene RRT-PCR (PCR), and virus re-isolation in 9–11 day old embryonated fowls' eggs [32] (two eggs per sample for each passage, with a total of two passages). Frozen tissues were thawed and a 10% homogenate prepared in PBS with antibiotics [14] using a powered homogeniser system (Omni homogenizer (GLH), using Omni Tip™ Plastic Generator Probes (hard tissue); Omni International, Marietta, GA, USA). Debris was removed by centrifugation (10 minutes at ∼1500 g) and the supernatant collected and passed through a 0.8 µm sterile filter (Satorius Ltd., Goettingen, Germany) prior to RNA extraction or virus re-isolation. Clarified material was then inoculated into embryonated fowls' eggs and the presence of virus determined by the haemagglutination of chicken erythrocytes [32]. The same tissue homogenate supernatant was used for the PCR assay (described below).

### Matrix (M) Gene RRT-PCR

Total RNA was extracted from swine clinical specimens (above) as previously described, including a positive extraction control that was prepared to yield a pre-determined Ct value [33]. The modified M gene RRT-PCR utilised the forward primer and probe described originally by Spackman *et al* [34] at final concentrations of 0.4 µM and 0.3 µM respectively, but the reverse primer consisted of an equimolar mixture of the original reverse primer plus a reverse primer modified to provide a perfect sequence match with the pandemic (H1N1) 2009 virus, typified by A/California/07/2009(H1N1)v (accession number: FJ966975). The four altered nucleotides in the modified reverse primer are indicated in upper case: 5′-tgc aaa Gac aCT ttc Cag tct ctg-3′. The final concentration of each of the reverse primers was 0.2 µM. Cycling conditions, temperatures and chemistry details were as outlined for the M gene RRT-PCR [35]. RNA extracted from a titrated allantoic fluid preparation of A/California/07/2009(H1N1)v was used to construct a ten-fold dilution series. This served to calibrate the Ct values derived from testing extracted clinical specimens by the modified M gene RRT-PCR with an equivalent viral infectivity titre [35] that is expressed in relative equivalent units (REU) [33].

### Histopathology and Immunohistochemistry

Following PME, all phosphate-buffered formalin-fixed samples were routinely embedded in paraffin wax. 4 µm thick sections, cut on a rotary microtome, were stained with haematoxylin and eosin or used for IHC detection of influenza A nucleoprotein by the avidin-biotin-peroxidase complex method [33] (see Protocol S1).

### Sequencing and Genetic Analyses

RNA was extracted from inoculum, swab or tissue samples (Table 2) using the QIAamp Viral RNA Kit (Qiagen). Genes were reverse transcribed and amplified using the OneStep RT-PCR Kit (Qiagen) (Primers available on request). RT-PCR products were excised from agarose gels and purified using the QIAquick Gel Extraction Kit (Qiagen). DNA was sequenced using the Prism BigDye Terminator v3.1 Cycle Sequencing Kit on the 3130 Genetic Analyzer (Applied Biosystems). All commercial kits were used following manufacturer's instructions. The Lasergene package (DNASTAR) was used for nucleotide sequence analysis and alignment. Full genome sequencing and further detailed genetic analyses were performed to compare the inoculum strain and selected output viruses (Table 2, Figure S6).

### Statistical Analyses

Descriptive data includes mean and standard deviation within the text. Mean and standard error of the mean are shown in graphical formats. Clinical scores, weights, temperatures and shedding levels were analysed using analysis of variance (one-way ANOVA with 95% confidence intervals) in the Minitab software package (Release 14).

## Supporting Information

Protocol S1 (0.03 MB DOC)

Text S1 (0.04 MB DOC)

Table S1 Clinical scoring system for influenza A challenge in pigs.
(0.03 MB DOC)

Figure S1 Study plan including timeline (date and day post-infection), animal numbers, post-mortem examinations (PME), transmission cycles (TC) and animal movements. An unplanned PME was carried out on pig 3261 dpi 17 on welfare grounds.
(0.07 MB DOC)

Figure S2 Mean daily rectal temperature (oC) plus standard errors for infected (INF), transmission cycle (TC) and control (C) pigs.
(0.07 MB DOC)

Figure S3 Mean live weight gain (kg) plus standard errors for infected (INF), transmission cycle (TC) and control (C) pigs.
(0.72 MB DOC)

Figure S4 Semi-quantitative nasal H1N1/09v virus shedding (REU log10; mean + SE) from infected (INF, panel A) and transmission cycle (TC, panels B–E) pigs. Panel F is the combined data (A–E).
(3.91 MB DOC)

Figure S5 Acute Phase Protein responses, mean C-Reactive protein (A) and Haptoglobin (B), for infected (INF) and transmission cycle (TC) pigs.
(0.03 MB DOC)

Figure S6 HA receptor selection. Figure shows a representative sequence chromatogram covering codons 225 and 226 (H3 numbering, [ref. 20]) for the inoculum, nasal swabs from directly infected pigs (INF) and contact exposure transmission cycle (TC1-4) isolates, and for two variations observed between middle lung lobe tissues at 1/2 dpi and 4/7 dpi. Amino acid and nucleotide sequences are shown above and below the trace respectively. Positions where a mixed amino acid population is observed are represented by an X.
(0.09 MB DOC)

## References

[1] Garten RJ, Garten RJ, Davis CT, Russell CA, Shu B (2009). Antigenic and genetic characteristics of swine-origin 2009 A(H1N1) influenza viruses circulating in humans.. Science.

[2] Smith GJ, Vijaykrishna D, Bahl J, Lycett SJ, Worobey M (2009). Origins and evolutionary genomics of the 2009 swine-origin H1N1 influenza A epidemic.. Nature.

[3] Brown IH, Klenk H-D, Matrosovich MN, Stech J (2008). The role of pigs in interspecies transmission, in: ‘Avian Influenza’.. Monogr Virol.;.

[4] Ma W, Lager KM, Vincent AL, Janke BH, Gramer MR (2009). The role of swine in the generation of novel influenza viruses.. Zoonoses and Public Health.

[5] Reid AH, Janczewski TA, Raina M, Lourens RM, Elliot AJ (2003). 1918 influenza pandemic caused by highly conserved viruses with two receptor-binding variants.. Emerg Infect Dis.

[6] Brown IH, Done SH, Spencer YI, Cooley WA, Harris PA (1993). Pathogenicity of a swine influenza H1N1 virus antigenically distinguishable from classical and Europeans strains.. Veterinary Record.

[7] Park AW, Wood JLN, Daly JM, Newton JR, Glass K (2004). The effects of strain heterology on the epidemiology of equine influenza in a vaccinated population.. Proc Biol Sci.

[8] Heegaard PMH, Pedersen HG, Jensen AL, Boas U (2009). A robust quantitative solid phase immunoassay for the acute phase protein C reactive protein in pig serum based on cytidine 5′-diphosphocholine coupled dendrimers.. J Immunol Methods.

[9] Heegaard PMH, Klausen J, Nielsen JP, González-Ramón N, Piñeiro M (1998). The porcine acute phase response to infection with *Actinobacillus pleuropneumoniae*. Haptoglobin, C-reactive protein, major acute phase protein and serum amyloid A protein are sensitive indicators of infection.. Comp Biochem Physiol.

[10] Olsen CW, Brown IH, Easterday BC, Van Reeth K, influenza Swine, Straw BE, Zimmerman JJ, D' Allaire S, Taylor DJ (2006). Diseases of Swine, 9th edition, Iowa State University Press, Ames, Iowa.

[11] Coates DM, Sweet C, Smith H (1986). Severity of Fever in Influenza: Differential Pyrogenicity in Ferrets Exhibited by H1N1 and H3N2 Strains of Differing Virulence.. J Gen Virol.

[12] Toms GL, Sweet C, Smith H (1977). Behaviour in ferrets of swine influenza virus isolated from man.. Lancet.

[13] Ottolini MG, Blanco JCG, Eichelberger MC, Porter DD, Pletneva L (2005). The cotton rat provides a useful small-animal model for the study of influenza virus pathogenesis,. J Gen Virol.

[14] Sorensen NS, Tegtmeier C, Andresen LO, Piñeiro M, Toussaint MJM (2006). The porcine acute phase protein response to acute clinical and subclinical experimental infection with *Streptococcus suis*.. Vet Immunol Immunopathol.

[15] OIE disease notification in pigs Canada (early May 2009) http://www.oie.int/wahis/public.php?page=single_report&pop=1&reportid=8065

[16] OIE disease notification in pigs Argentina (early June 2009). http://www.oie.int/wahis/public.php?page=single_report&pop=1&reportid=8227

[17] OIE (2009) WAHID Interface – Animal Health Information, Vol. 22 - No. 33, 13 Aug, 2009. Influenza A subtype H1N1, Australia. Follow-up report No. 1 (Final report), 13/08/09. http://www.oie.int/wahis/public.php?page=single_report&pop=1&reportid=8358

[18] Streta D, Kedkovid R, Tuamsang S, Kitikoon P, Thanawongnuwech R (2009). Pathogenesis of swine influenza virus (Thai isolates) in weanling pigs: an experimental trial.. Virol J.

[19] Weingartl HM, Albrecht RA, Lager KM, Babiuk S, Marszal P (2009). Experimental infection of pigs with the human 1918 pandemic influenza virus.. J Virol.

[20] Itoh Y, Shinya K, Kiso M, Watanabe T, Sakoda Y (2009). In vitro and in vivo characterization of new swine-origin H1N1 influenza viruses.. Nature.

[21] Lange E, Kalthoff D, Blohm U, Teifke JP, Breithaupt A (2009). Pathogenesis and transmission of the novel swine origin influenza virus A/H1N1 after experimental infection.. J Gen Virol.

[22] Lipatov AS, Kwon YK, Sarmento LV, Lager KM, Spackman E (2008). Domestic pigs have low susceptibility to H5N1 highly pathogenic avian influenza viruses.. PLoS Pathog.

[23] Kida H, Ito T, Yasuda J, Shimizu Y, Itakura C (1994). Potential for transmission of avian influenza viruses to pigs.. J Gen Virol.

[24] Van Reeth K, Braeckmans D, Cox E, Van Borm S, van den Berg T (2009). Prior infection with an H1N1 swine influenza virus partially protects pigs against a low pathogenic H5N1 avian influenza virus.. Vaccine.

[25] Munster VJ, de Wit E, van den Brand JM, Herfst S, Schrauwen EJ (2009). Pathogenesis and Transmission of Swine-Origin 2009 A(H1N1) Influenza Virus in Ferrets.. Science.

[26] Maines TR, Jayaraman A, Belser JA, Wadford DA, Pappas C (2009). Transmission and Pathogenesis of Swine-Origin 2009 A(H1N1) Influenza Viruses in Ferrets and Mice.. Science.

[27] Matrosovich MN, Gambaryan AS, Klenk H-D (2008). Receptor specificity of influenza viruses and its alteration during interspecies transmission..

[28] Mochalova L, Gambaryan A, Romanova J, Tuzikov A, Chinarev A (2003). Receptor-binding properties of modern human influenza viruses primarily isolated in Vero and MDCK cells and chicken embryonated eggs.. Virology.

[29] Matrosovich MN, Matrosovich TY, Gray T, Roberts NA, Klenk H-D (2004). Human and avian influenza viruses target different cell types in cultures of human airway epithelium.. Proc Natl Acad Sci U S A.

[30] Gambaryan AS, Karasin AI, Tuzikov AB, Chinarev AA, Pazynina GV (2005). Receptor-binding properties of swine influenza viruses isolated and propagated in MDCK cells.. Virus Research.

[31] Tumpey TM, Maines TR, Van Hoeven N, Glaser L, Solórzano A (2007). A two-amino acid change in the hemagglutinin of the 1918 influenza virus abolishes transmission.. Science.

[32] OIE (World Organisation for Animal Health). Swine Influenza: Manual of Diagnostic Tests and Vaccines for Terrestrial Animals (2008). http://www.oie.int/Eng/Normes/Mmanual/2008/pdf/2.08.08_SWINE_INFLUENZA.pdf.

[33] Löndt BZ, Nunez A, Banks J, Nili H, Johnson LK (2008). Pathogenesis of highly pathogenic avian influenza A/turkey/Turkey/1/2005 H5N1 in pekin ducks (*Anas platyrhynchos*) infected experimentally.. Avian Pathology.

[34] Spackman E, Senne DA, Myers TJ, Bulaga LL, Garber LP (2002). Development of a real-time reverse transcriptase PCR assay for type A influenza virus and the avian H5 and H7 haemagglutinin subtypes.. Journal of Clinical Microbiology.

[35] Slomka MJ, Pavlidis T, Coward V, Voermans J, Koch G (2009). Validated RealTime reverse transcriptase PCR methods for the diagnosis and pathotyping of Eurasian H7 avian influenza viruses.. Influenza and Other Respiratory Viruses.

