# Supplementary material for: Replication, Pathogenesis and Transmission of Pandemic (H1N1) 2009 Virus in Non-Immune Pigs
Source: PLoS One. 2010 Feb 5;5(2):e9068. doi: 10.1371/journal.pone.0009068 (PMC2816721; doi:10.1371/journal.pone.0009068)
Supplement: Protocol S1 — (0.03 MB DOC) [file pone.0009068.s001.doc]

**Supporting Protocol**

**Acute Phase Protein (APP) assays.** The serum concentrations of the acute phase proteins, C-reactive protein and haptoglobin were determined by ELISA methods. C-reactive protein was analysed by a sandwich type ELISA using dendrimer-coupled cytidine diphosphocholine (a C-reactive protein-binding ligand) in the coating layer (as described in ref. 8) employing polyclonal rabbit anti-human antibodies with cross-reactivity towards porcine C-reactive protein followed by peroxidase-conjugated goat anti rabbit antibody for detection (both antibodies from DAKO, Glostrup, Denmark). The cross-reactivity of the anti human C-reactive protein antibody with pig C-reactive protein was demonstrated previously [28] and combining it with catching of C-reactive protein by diphosphocholine ensured specificity for C-reactive protein. Pooled pig serum calibrated against a human C-reactive protein calibrator (DAKO A0073) was used as standard. The detection limit was 0.035 mg/L (human equivalents). Haptoglobin was determined by a sandwich ELISA using an in-house mouse anti-porcine haptoglobin monoclonal antibody in the coating layer, and biotinylated commercial rabbit anti-human haptoglobin (DAKO A0030) as the detection antibody as described [9] with a detection limit of 33 mg/L (porcine haptoglobin equivalents). All of the ELISAs described above were developed using TMB Plus ready-to-use substrate from Kem-En-Tec (Denmark) containing 3,3’,5,5’-tetramethylbenzidine and hydrogen peroxide. Development of colour was stopped with 0.5 M sulphuric acid and optical densities of wells were read at 450 nm subtracting non-specific coloration at 650 nm using an automatic plate reader (Thermo Multiskan Ex spectrophotometer, Thermo Scientific, Waltham, MA, USA). All samples including standards were determined in duplicate. Sample values were calculated from the curve fitted to the readings of the standard (using Ascent software v. 2.6, Thermo Scientific).

**Immunohistochemistry (IHC).** All buffered formalin fixed samples were routinely embedded in paraffin wax. 4 µm thick sections, cut on a rotary microtome, were de-waxed in xylene and dehydrated in absolute alcohol before being immersed for 15 minutes in a hydrogen peroxide/methanol block (30ml 100 vol H_2_0_2_/1000ml methanol, VWR, Leicester, UK) to quench endogenous peroxide activity and treated with Protease XXIV for 10 minutes at room temperature. Slides were assembled into Shandon coverplates to facilitate IHC using the Shandon Sequenza system (Shandon, USA) and primary antibody cross-reactivity with tissue constituents was prevented using a normal immune serum block for 30 min (Vector Laboratories). Samples were subsequently incubated at room temperature with a mouse anti-influenza A nucleoprotein monoclonal antibody (Statens Serum Institute, Denmark) for 1 hour, biotinylated Goat anti-mouse Fab fragment secondary antibody (Jackson Immunoresearch, Baltimore, USA) for 30 minutes and avidin-biotin-peroxidase conjugate (***^elite^*** Vector laboratories) for 30 min. Non-specific binding was removed with three successive washes with 0.005M TBS, pH7.6 0.85% NaCl after each incubation. The immunohistochemical signal was visualised using 3,3 diaminobenzidine (Sigma-Aldrich) and sections were counterstained in Mayer’s haematoxylin (Surgipath, Peterborough, UK), dehydrated in absolute alcohol, cleared in xylene and coverslipped using Dibutyl Phthalate Xylene (DPX) and glass coverslips. Protein concentration-matched mouse immunoglobulin isotype antibody (Vector Laboratories, Peterborough, UK) were included as technique controls.
