# Supplementary material for: Replication, Pathogenesis and Transmission of Pandemic (H1N1) 2009 Virus in Non-Immune Pigs
Source: PLoS One. 2010 Feb 5;5(2):e9068. doi: 10.1371/journal.pone.0009068 (PMC2816721; doi:10.1371/journal.pone.0009068)
Supplement: Text S1 — (0.04 MB DOC) [file pone.0009068.s002.doc]

**Supporting Text**

# Clinical signs. The mean (SD) rectal temperature in all uninfected pigs (control and contact exposed animals prior to their inclusion in each of TC1 to TC4; n=127) over the course of the study (Fig. S2) was 37.9^o^C (+0.96^o^C), 38.3^o^C (+0.77^o^C) in the contact exposed pigs during and following the four transmission cycle phases (n=126), and 38.7^o^C (+0.96^o^C) in infected animals (n=93), giving highly significant differences between the overall mean temperatures of these three categories of study animals (P<0.001). The difference between contact exposed (TC1-TC4) and uninfected (as above) animals was also significant (P=0.003). Daily comparisons demonstrated that the rectal temperature of the infected pigs was significantly higher than that of the TC and control animals for dpi/dpc 1, 2, 4 (P<0.05, P range 0.002-0.021), 5* (*10% level, P=0.083) and 6 (P<0.05). In addition, the mean rectal temperature in the contact exposed pigs was also significantly greater (P=0.036) than that in the other pigs at dpc 7 (Fig. S2).

Variations were also apparent in the overall peak and intervals to peak rectal temperatures recorded for the contact exposed pigs during TC1, TC2 and TC3, which were generally lower and occurred later (6-8 days post-contact) than those recorded in the infected pigs. Similarly, the peak rectal temperature during TC4 was comparable to that recorded for the pigs in TC1-TC3, but the interval to and duration of peak rectal temperature was equivalent to that recorded for the infected pigs.

The mean clinical score per day for the infected pigs peaked at dpi 4-6. Although there was no overt mortality, it was possible that 3/19 (~15%) pigs may have succumbed according to expert veterinary opinion. Progressive clinical deterioration suggested that two (3244, 3247) of the three pigs may not have recovered if planned post mortem examination had not taken place (Fig. S1). In addition, one pig (3260) was humanely killed on welfare grounds at dpi 17 due to severe diarrhoea and weight loss. Whilst full recovery was evident in the second remaining infected animal after dpi 7 and those in TC1. Scheduled PME of the remaining contact exposed pigs (TC2-TC4) resulted in their removal from the study (Fig. S1) prior to complete recovery. The mean (SD) clinical score per day post-exposure was greater in the infected animals than that in the TC pigs, and the overall mean score was higher (1.67+1.15) than that of the TC animals (1.59+0.76), but not significant. However, a significant difference in mean clinical score per day post-exposure was evident at dpi 6 compared with dpc 6 (3.25 *vs*. 1.8; P<0.001).

**Daily Live Weight Gain (LWG).** The animals were randomised prior to the start of the study. The mean starting body weight per category was 7.9kg for the uninfected controls, 6.8kg for the infected group and, 8.2kg for the contact exposed animals (TC1-TC4). The age of the contact exposed animals at the start of each TC was greater than the controls and infected categories by 2, 5, 9 and 15 days for TC1-TC4 respectively (as per the Study Plan, Fig. S1). Weight loss was limited (~5%), occurring in 3/3 directly infected animals between dpi 6-8 and in 5/8 contact exposed animals between dpc 2-16. The mean daily live weight gain (SD) over the course of the study in each of the study categories was 0.14+0.32kg in infected animals (n=81), 0.31+0.46kg in TC animals (n=126), and 0.15+0.63kg in control animals (n=106). The TC animals gained significantly (P=0.014) more weight per day than both the control and infected animals, which we suggest is a function of the combined effects of increased age and bodyweight at the onset of each TC. Daily weight gain comparisons between the three groups (INF, TC and Controls) were significantly different (P<0.05) from dpi/dpc 0-6, 8 and 11, or substantial (P range 0.05-0.10) from dpi/dpc 7, 9,10, 14 and 15, with the main contributing group being the TC animals, no significant difference was detected at dpi/dpc 12, 13 or 16-19, mainly influenced by the decreasing groups sizes (Fig. S3).

Using age-matched animals for each group (n=2), daily live weight gain was not significantly different (P=0.225). Infected pigs (n=40) gained the least weight per day 0.09+0.34kg, TC pigs (n=40) gained 0.28+0.51kg daily, whilst control animals (n=42) gained 0.28+0.73kg per day.

Therefore, infection with pandemic (H1N1) 2009 virus affects daily live weight gain negatively, and hence will delay the time taken for pigs to reach market weights, with potential economic impacts.

**Virus infection dynamics: Shedding patterns and routes**

**Nasal shedding.** The mean shedding level from the nasal cavity per day in the infected group did not vary significantly from one animal to the next (P>0.05); the minimum daily mean was 0.98 REU log_10_, and the maximum daily mean was 2.62 REU log_10_ (range at individual level 0.0 - 4.6 REU log_10_). The mean shedding level from the nasal cavity per day in the four TC groups did not vary significantly from one animal to the next (P>0.05); the minimum daily mean was 0.95 REU log_10_ and the maximum daily mean was 1.52 REU log_10_ (range at individual level 0.0 - 5.0 REU log_10_). Comparing between the four TC groups there was no significant difference (P>0.05) in the mean daily shedding levels from the nasal cavity.

Comparisons of the total mean daily nasal shedding between all the infected animals and the contact exposed pigs in groups TC1 to TC4 combined shows that the levels of nasal shedding in infected pigs (1.57 REU log_10_) at the group level were substantially higher (significant at the 10% level, P=0.073) than those in the four TC groups (1.22 REU log_10_) combined (Fig.1A). Comparisons at the daily level indicated that there was no significant difference in the level of nasal shedding between infected and TC animals with the exception of three days. At dpc 4 and 7 the TC pigs shed significantly (P= 0.03 and P=0.05 respectively) more virus from the nasal passages than the directly infected group.

In conclusion, irrespective of whether the pigs were directly infected with virus or became infected by contact transmission at the individual level they shed similar levels of virus via the nasal cavity (Fig. 1A and Fig. S4).

**Oral shedding.** The oral shedding profiles (mean) for infected and TC groups (1-4) were similar, with detectable levels found at day 3 (dpi/dpc), peaking at day 4-6, and declining significantly by day 7-8. Peak oral shedding from directly infected animals (2.9 REU log_10_) was detected 1-2 days later than that from TC animals, and detectable oral shedding had ceased by dpi 8. In all animals that went beyond dpi 3 (n=14) viral RNA could be detected in the oral cavity, with a longer duration of sustained, trace levels of shedding from the oral cavity of the contact exposed animals until day 16 (Fig. 1B). Overall, there was no significant difference in the levels of oral shedding between the infected animals and the pairs of contact animals (TC1-TC4), or between the different TC groups. Comparisons at the daily level indicated that there was no significant difference in the level of oral shedding between infected and TC animals with the exception of three days. At dpc 4 TC pigs shed significantly (P=0.027) more virus than the dpi 4 animals. At dpi 5 and 6 the INF pigs shed significantly (P= 0.047 and P=0.022 respectively) more virus from the oral route than the in-contact groups.

**Ocular shedding.** The ocular shedding profiles (mean) for infected and TC groups (1-4) were also very similar to each other (Fig. 1C), but peak shedding levels were at least 1 REU log_10_ lower compared with the oral shedding levels. From both infected and contact exposed pigs, ocular shedding was detectable as early as day 1-2 (dpi/dpc), peaking at days 5-6 (mean ~1 REU log_10_), with sporadic detections after day 8 from TC (day 9-10) and infected (day 13) animals, after which shedding ceased. Intermittent ocular shedding was detected in all but one of the animals (TC3, 3259) that went beyond dpi 4 (n=12), and individual animal variations in levels of ocular shedding were apparent (0.0 REU – 2.9 REU log_10_). There was no significant difference, overall or at a daily comparison level, between the infected animals in terms of the level of virus shedding from the ocular route, or between the infected animals and the pairs of contact exposed animals (TC1-TC4). However, there were significant differences between the TC animals, with ocular shedding levels from two individuals (TC1, 3254 and TC2, 3256) greater than from the other TC pigs (P=0.029).

**Gross pathology.** At PME of two infected pigs (INF, 3249 and 3252) on dpi 2, acute catarrhal rhinitis was observed, but no gross lesions were found in the lungs. A small number of macroscopic pulmonary lesions, restricted to apical and middle lung lobes, and consisting of multifocal areas of lobular consolidation with red firm appearance, and clearly demarcated from surrounding parenchyma, were observed at dpi 3 and dpi 4. Moderate lymphadenopathy of the tracheobronchial and mediastinal lymph nodes was also observed. Similar lesions, but more extensive and affecting, on occasions, almost entire pulmonary lobes, were observed at PME of infected pigs on dpi 7 (n=2, 3244 and 3260). One of the animals (3260) also displayed a severe mucopurulent rhinitis. The presence of similar pulmonary changes was observed affecting more than 70% of the apical, middle and accessory lung lobes of one (3257) of the in-contact pigs (TC4) subject to PME on dpc 11 (the final day of the study). No significant gross changes were observed at PME of the remaining infected pigs on dpi 17 (3261) and dpi 21 (3245).

**Histopathology.** Acute rhinitis was consistently observed in the nasal cavity of infected pigs at dpi 2 (n=2), but no other significant changes were observed in any of the other tissues examined. Focal, acute, necrotising bronchiolitis and peribronchiolar mononuclear infiltration and alveolitis of lobular distribution was observed at dpi 3 in the right middle lung lobe. Similar changes were observed at dpi 4, but affecting multiple lung lobes. The affected bronchioles showed mild attenuation of the epithelial layer and their lumen was occupied with plugs of cells debris and leukocytes (polymorphic nucleated cells and macrophages) completely or partially blocking the lumen. Peribronchiolar mononuclear (lymphohistiocytic) infiltration was observed in conjunction with a moderate thickening of the alveolar septae in the surrounding area of pulmonary parenchyma due to cellular infiltration. The severity and extension of these lesions was increased at dpi 7, and in addition, multiple bronchioles presented with epithelial hyperplasia. Histopathology of tissues from the infected pigs examined at dpi 17 and dpi 21 revealed no, or only minimal, residual lesions in lung lobules previously affected, characterised by occasional lymphoplasmacytic peribronchiolar infiltration and focal areas of bronchointerstitial pneumonia.

The histopathological changes observed in the lungs of the TC4 in-contact, animal (3257, dpc 11) were those of a moderate, bronchointerstitial pneumonia, with lymphohistiocytic infiltration in alveolar septae, and bronchiolar epithelium hyperplasia.

# Immunohistochemistry (IHC). Detection of viral antigen by IHC (Table 1) was observed profusely in the epithelial cells of the nasal turbinates at dpi 2 (Fig. 2), and focally in bronchiolar epithelial cells and a small number of alveolar macrophages (AM) and interstitial macrophages in the right middle lung lobe of one of the animals, despite the absence of morphological changes. Immunolabelling was more intense within the nucleus, but was also cytoplasmic (as expected when labelling influenza A nucleoprotein). A similar distribution was observed at dpi 3 affecting a higher number of epithelial cells and bronchioles, and also present in the cell debris of bronchiolar plugs. Morphological features of the immunolabelled cells in the alveoli were mainly consistent with AM. At dpi 4, extensive respiratory tract immunolabelling was observed, comprising respiratory epithelial cells in the turbinates, cervical and thoracic trachea, olfactory epithelium and nasopharynx, multifocally in bronchioles and pulmonary macrophages in the lung, and also in epithelial cells of large diameter bronchi. At dpi 7, a similar distribution was observed in animal 3260 in a larger number of lobules. However, minimal amount of viral antigen was detected in animal 3244, despite marked mononuclear alveolitis, and it was restricted to AM. No viral antigen was detected at dpi 17 and dpi 21 (3261 and 3245) indicating viral clearance. Immunolabelling in the TC4 animal (3257, dpc 11) was restricted to cellular debris present in the bronchiolar plugs of affected lobules, but not observed in epithelial cells or pulmonary macrophages.

# Sequencing and Genetic Analyses. In addition to the described receptor binding mutations in HA1, a mutation was observed in HA2 at codon 54. The inoculum had 54_2_S which altered to 54_2_F in pig 3254 (TC1), and then a mixed population of F/S emerged in pig 3256 (TC2) with reversion back to 54_2_S in TC3. No other H1N1/2009 virus appears to have this mutation (500 full length HA sequences, data accessed 15^th^ November 2009 from NCBI Influenza Virus Resource, [36]), suggesting that it could have been selected during the pig experiment. Codon 54_2_ is in the HA2 coiled-coil domain and therefore may have an affect on pH activation of the HA protein. The mutation S54_2_R/K in an H5 isolate resulted in significant increases in HA cell surface expression [37,38]. It is unclear what affect (if any) S54_2_F would cause. However, since reversion was observed back to 54_2_S it is assumed this mutation was not favourable.

**References**

36. Bao Y, **Bolotov**, P, **Dernovoy**, D, **Kiryutin**, B, **Zaslavsky**, L, *et al*. The Influenza Virus Resource at the National Center for Biotechnology Information. *J. Virol*. **82**, 596-601 (2008).

37. Steinhauer, D. A, [Martín](http://www.pnas.org/search?author1=Javier+Martín&sortspec=date&submit=Submit), J, Lin, Y, Wharton, S, Oldstone, M, *et al.* Studies using double mutants of the conformational transitions in influenza haemagglutinin required for its membrane fusion activity. *Proc. Natl. Acad. Sci. USA,* **93,** 12873-8 (1996)

38. Reed, L, Yen, H, DuBois, R, Bridges, O, Salomon, R, *et al.* Amino acid residues in the fusion peptide pocket regulate the pH of activation of the H5N1 Influenza virus haemagglutinin pocket. *J. Virol.* **83**, 3568-80 (2009).
