# Supplementary material for: Replication, Pathogenesis and Transmission of Pandemic (H1N1) 2009 Virus in Non-Immune Pigs
Source: PLoS One. 2010 Feb 5;5(2):e9068. doi: 10.1371/journal.pone.0009068 (PMC2816721; doi:10.1371/journal.pone.0009068)
Supplement: Table S1 — Clinical scoring system for influenza A challenge in pigs. (0.03 MB DOC) [file pone.0009068.s003.doc]

**Table S1 Clinical Scoring System for Influenza A Challenge in Pigs**

| Clinical Signs | **Score** |
| --- | --- |
| - Normal behaviour | 0 |
| - Lethargic - Sneeze/cough - Increasing temperature | 1 |
| - Up to 5% weight loss - Pyrexia >39.5^o^C single day - Upper respiratory tract discharge (eyes/nose/mouth) - Reduced activity - Observed to be eating/drinking | 2 |
| - 5-10% weight loss - Pyrexia >40.0^o^C or >1-2 days - Ataxia - Onset of breathing difficulties | 3 |
| - 15% weight loss - Sustained pyrexia >2 days  Unwilling to eat/drink  - Persistent breathing difficulties/distress | 4 |
| - 20% weight loss - Prostrate/will not respond when stimulated - Unable to eat/drink | 5 |
| - Found dead | 6 |

Humane end point: score of >4.
