# Supplementary material for: Replication, Pathogenesis and Transmission of Pandemic (H1N1) 2009 Virus in Non-Immune Pigs
Source: PLoS One. 2010 Feb 5;5(2):e9068. doi: 10.1371/journal.pone.0009068 (PMC2816721; doi:10.1371/journal.pone.0009068)
Supplement: Figure S2 — Mean daily rectal temperature (oC) plus standard errors for infected (INF), transmission cycle (TC) and control (C) pigs. (0.07 MB DOC) [file pone.0009068.s005.doc]

**Rectal Temperature**

34.0

35.0

36.0

37.0

38.0

39.0

40.0

41.0

0

1

2

3

4

5

6

7

8

9

10

11

12

13

14

15

16

17

18

19

20

21

**dpi / dpc**

**Temperature (^o^C)**

INF-Mean

TC-Mean

C-Mean
