# Supplementary material for: Replication, Pathogenesis and Transmission of Pandemic (H1N1) 2009 Virus in Non-Immune Pigs
Source: PLoS One. 2010 Feb 5;5(2):e9068. doi: 10.1371/journal.pone.0009068 (PMC2816721; doi:10.1371/journal.pone.0009068)
Supplement: Figure S4 — Semi-quantitative nasal H1N1/09v virus shedding (REU log10; mean + SE) from infected (INF, panel A) and transmission cycle (TC, panels B–E) pigs. Panel F is the combined data (A–E). (3.91 MB DOC) [file pone.0009068.s007.doc]

A

B

C

D

E

F
