# Supplementary figures and images for: Replication, Pathogenesis and Transmission of Pandemic (H1N1) 2009 Virus in Non-Immune Pigs
Source: PLoS One. 2010 Feb 5;5(2):e9068. doi: 10.1371/journal.pone.0009068 (PMC2816721; doi:10.1371/journal.pone.0009068)

Inoculum

Direct infection

Transmission

Mid-lung tissues

1 and 2 dpi

4 and 7 dpi

**225 226**

**225 226**

**225 226**

**225 226**


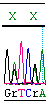

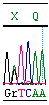

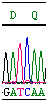

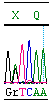

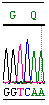


**225 226**


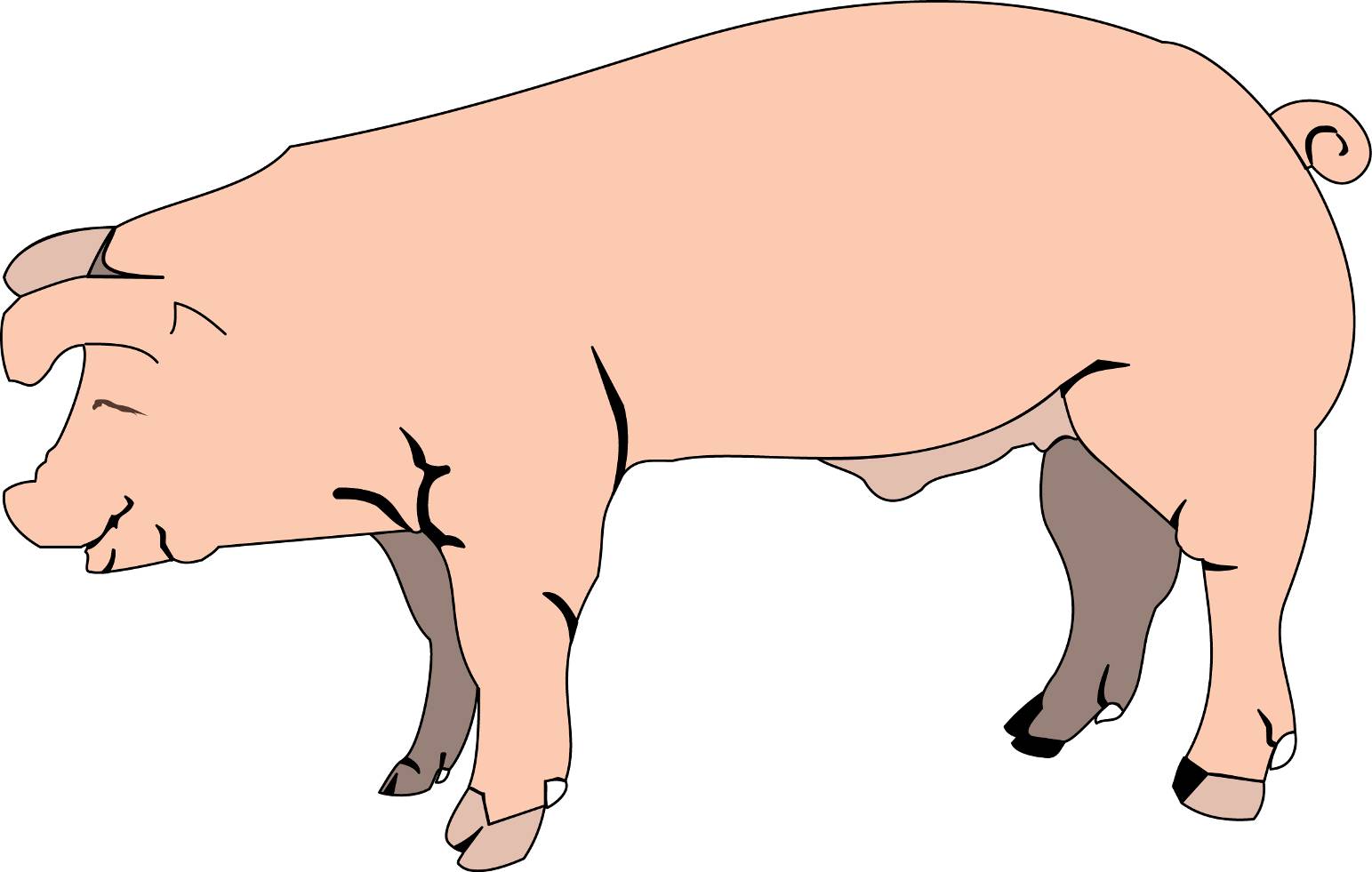

Supplement: Figure S6 — HA receptor selection. Figure shows a representative sequence chromatogram covering codons 225 and 226 (H3 numbering, [ref. 20]) for the inoculum, nasal swabs from directly infected pigs (INF) and contact exposure transmission cycle (TC1-4) isolates, and for two variations observed between middle lung lobe tissues at 1/2 dpi and 4/7 dpi. Amino acid and nucleotide sequences are shown above and below the trace respectively. Positions where a mixed amino acid population is observed are represented by an X. (0.09 MB DOC) [file pone.0009068.s009.doc]
