# Supplementary material for: Replication, Pathogenesis and Transmission of Pandemic (H1N1) 2009 Virus in Non-Immune Pigs
Source: PLoS One. 2010 Feb 5;5(2):e9068. doi: 10.1371/journal.pone.0009068 (PMC2816721; doi:10.1371/journal.pone.0009068)
Supplement: Supplementary file 7 [file pone.0009068.s004.doc]

		Infected (Inf) Pigs nH1N1 (n=11)		Contact Transmission Cycle (TC) Pigs (n=8)				Control pigs (n=3)	
n=22									
Day / Date		Room 1	dpi		Room 2	Room 3	Room 4		
Monday	11/05/2009	Arrive	-4						
Tuesday	12/05/2009	*Daily Veterinary inspections, swabbing, weights, temperature (& pre-bleed on dpi -3)*							
Wednesday	13/05/2009		-2						
Thursday	14/05/2009		-1						
Friday	15/05/2009	**Infect I/N (n=11)**	0	TC1	TC2	TC3	TC4		
Saturday	16/05/2009	1x PME-inf 3253	1						
Sunday	17/05/2009	2x PME-inf 3252 & 3249	2	**2x Contact (2C) pigs into Room 1** 3240 & 3254					
Monday	18/05/2009	2x PME-inf 3246 & 3251	3						
Tuesday	19/05/2009	2x PME-inf 3243 & 3248	4						
Wednesday	20/05/2009		5		**2C + 2 naïve (2n) pigs into Room 2** 3250 & 3256				
Thursday	21/05/2009		6						Mock infected pigs (n=2) PME day 3 & day 20, 3242 & 3241
Friday	22/05/2009	2x PME-inf 3244 & 3260	7					Control pigs (n=1) 3255	
Saturday	23/05/2009		8			**2n + 2 new naïve (2nn) pigs into Room 3** 3258 & 3259			
Sunday	24/05/2009		9						
Monday	25/05/2009		10						
Tuesday	26/05/2009		11						
Wednesday	27/05/2009		12						
Thursday	28/05/2009		13						
Friday	29/05/2009		14				**2nn + 2 pigs (2p) into clean Room 2** 3247 & 3257		
Saturday	30/05/2009		15						
Sunday	31/05/2009		16						
Monday	01/06/2009	1xPME-inf 3261	17						
Tuesday	02/06/2009		18						
Wednesday	03/06/2009		19						
Thursday	04/06/2009		20					2xPME - C & M	
Friday	05/06/2009	1xPME-inf 3245	21	4xPME - TC1 & TC2 (dpc 16 & 19)					
Saturday	06/06/2009		22						
Sunday	07/06/2009		23						
Monday	08/06/2009		24						
Tuesday	09/06/2009		25			4xPME - TC3 & TC4 (dpc 11 & 16)			
